# Draft Genome Sequence of a Cold-Adapted *Pseudomonas* sp. Strain, BGI-2, Isolated from the Ice of Batura Glacier, Pakistan

**DOI:** 10.1128/MRA.00320-19

**Published:** 2019-05-09

**Authors:** Pervaiz Ali, Aamer Ali Shah, Fariha Hasan, Haiyuan Cai, Ana Sosa, Feng Chen

**Affiliations:** a Institute of Marine and Environmental Technology, University of Maryland Center for Environmental Science, Baltimore, Maryland, USA; b Applied Environmental and Geomicrobiology Laboratory, Department of Microbiology, Quaid-i-Azam University, Islamabad, Pakistan; c Nanjing Institute of Geography and Limnology, Chinese Academy of Sciences, Nanjing, China; Indiana University, Bloomington

## Abstract

*Pseudomonas* sp. strain BGI-2 is a psychrotrophic bacterium isolated from the ice of Batura Glacier in the Karakoram mountain range. This strain produces a high yield of exopolysaccharide (EPS) at low temperatures and exhibits high freeze-thaw tolerance. The BGI-2 genome contains 11 EPS-producing genes, which are not found in the closely related *Pseudomonas* strains.

## Abstract (precis)

*Pseudomonas* sp. strain BGI-2 is a psychrotrophic bacterium isolated from the ice of Batura Glacier in the Karakoram mountain range.

## ANNOUNCEMENT

Cold habitats have been successfully colonized by microorganisms known as psychrophiles or psychrotrophs, making them the most abundant extremophiles in terms of diversity, distribution, and biomass (1). Their successful colonization of harsh cold environments is the result of molecular evolution and adaptations (2). The production of cryoprotectants such as exopolysaccharide is one of the key strategies used by microorganisms to withstand the damage caused by freezing conditions (3 – 5). *Pseudomonas* is a genus of the class *Gammaproteobacteria* known for its metabolic versatility and ability to inhabit diverse environments, including the extremes (6). Cold-adapted *Pseudomonas* species have been isolated from different cold environments, including polar and nonpolar regions (7, 8). *Pseudomonas* sp. strain BGI-2 was isolated from the ice of Batura Glacier using R2A medium (Difco). The strain is halotolerant, with wide growth ranges for temperature (4 to 35°C) and pH (5 to 11).

A pure culture of BGI-2 was grown in R2A broth at 15°C, and the genomic DNA was extracted from an overnight culture using an UltraClean microbial DNA isolation kit (Mo Bio Laboratories). The concentration and purity of the extracted DNA were determined using a NanoDrop 2000 spectrophotometer (Thermo Fisher Scientific). The DNA was sequenced using Illumina MiSeq sequencing. The library was prepared using a Nextera XT DNA library prep kit (Illumina, Inc., San Diego, CA), according to the manufacturer’s protocol, and sequencing was performed in a MiSeq 2 × 250-bp run. Raw reads were processed for quality trimming and adapter removal using Trimmomatic v.0.33 (9). *De novo* assembly of the processed reads was performed using SPAdes v.3.10.0 (10) with default settings to yield 106 contigs. The draft genome sequence of *Pseudomonas* sp. strain BGI-2 consists of 6,267,352 bp with a GC content of 58.9% and an *N*_50_ value of 110,913 bp. The mean read coverage for the assembly was 158.0×. The Rapid Annotations using Subsystems Technology (RAST) v.2.0 server (11) and the NCBI Prokaryotic Genome Annotation Pipeline (PGAP) v.4.7 (12) were used for annotation of the assembled contigs. This resulted in the identification of a total of 6,075 genes comprising 5,566 protein-coding genes, 73 RNAs, 60 tRNAs, and 376 pseudogenes.

Overall genome relatedness indices (OGRI) (13) between BGI-2 and the most closely related *Pseudomonas* species were calculated (Table 1). Digital DNA-DNA hybridizations (dDDH) were determined online using the Genome-to-Genome Distance Calculator (GGDC) (14). The estimated DDH values were calculated using formula 2 at the GGDC website (13, 14). The average nucleotide identity (ANI) was calculated using the server-based software EzBioCloud (15). *Pseudomonas* sp. strain BGI-2 was most closely related to Pseudomonas kilonensis DSM 13647, with ANI and dDDH values of 83.13% and 27.3%, respectively (Table 1). The proposed ANI and dDDH values for species boundaries are 95 to 96% and 70%, respectively (16, 17). These values are below the accepted threshold for species demarcation, suggesting that *Pseudomonas* sp. strain BGI-2 could be a novel species in the genus *Pseudomonas*.

**TABLE 1:** dDDH and ANI values for *Pseudomonas* sp. strain BGI-2 compared to those of closely related *Pseudomonas* strains based on the 16S rRNA gene sequence similarity from EzBioCloud

*Pseudomonas* strain	GenBank accession no.	16S rRNA gene similarity (%)	dDDH (%)	ANI (%)
P. caspiana FBF102	LOHF00000000	99.45	22.7	77.77
P. amygdali CFBP 3205	JYHB00000000	99.32	22.6	77.99
P. cerasi 58	LT222319	99.18	22.6	78.20
P. congelans DSM 14939	FNJH00000000	99.18	22.5	78.19
P. kilonensis DSM 13647	LHVH00000000	99.18	27.3	83.13
P. syringae KCTC 12500	AYTM00000000	99.11	22.7	77.98
P. fluorescens DSM 50090	LHVP00000000	98.56	24.8	81.10

The *Pseudomonas* sp. strain BGI-2 genome contains stress response genes which are responsible for osmotic stress, oxidative stress, cold shock, detoxification, and carbon starvation. Interestingly, 11 EPS-producing genes were identified in the BGI-2 genome, while none of the 7 mesophilic *Pseudomonas* species in Table 1 contain these genes. The EPS genes are EpsE (undecaprenyl-phosphate galactosephosphotransferase), CpsA (capsular polysaccharide synthesis enzyme CpsA, sugar transferase), CpsB (capsular polysaccharide synthesis enzyme CpsB), CpsC (capsular polysaccharide synthesis enzyme CpsC, polysaccharide export), CpsD (capsular polysaccharide synthesis enzyme CpsD, exopolysaccharide synthesis), and 3 genes each of Glt1 (glycosyltransferase, group 1 family protein) and Glt2 (glycosyltransferase , group 2 family protein). Production of exopolysaccharide by microorganisms is considered an adaptation to survive freezing environments (3, 4). Further in-depth study of the genomic data will help us understand the molecular basis of cold adaptation.

### Data availability.

The draft genome sequence has been deposited in NCBI GenBank under the accession number SISB00000000, 16S rRNA gene sequence accession number MH681214, BioProject number PRJNA523205, and BioSample number SAMN10966221. The raw reads have been deposited in the NCBI Sequence Read Archive (SRA) with the accession number SRR8715451.
